# Home ranges and movements of resident graylag geese (*Anser anser*) in breeding and winter habitats in Bavaria, South Germany

**DOI:** 10.1371/journal.pone.0202443

**Published:** 2018-09-17

**Authors:** Anke Kleinhenz, Andreas Koenig

**Affiliations:** Technical University of Munich, Wildlife Biology and Management Unit, Freising, Germany; Sichuan University, CHINA

## Abstract

In several countries in northwestern Europe, the number of resident greylag geese (*Anser anser*) has increased by up to 20% a year over the last few decades. The increasing numbers of geese are causing problems in tourism and agriculture. The number of resident greylag geese visiting the Altmuehlsee lake, a recreational area in the south of Germany, has also increased, especially in June and July. However, the geese leave the lake again at the beginning of August. 30 adult greylag geese were banded with telemetry transmitters during molting at the Altmuehlsee lake to determine when the geese leave the lake in August and what structures they need in their winter habitat. The distances the greylag geese flew from their resting sites to foraging sites were calculated, as well as the size of their home ranges and the proportions of each habitat type in the home range. It was found that the greylag geese used small home ranges while rearing their young, and fed close to the water. After molting, the size of their home ranges increased. They fed on fields and meadows around the lake. In the winter habitat, the geese also had a small home range, and they preferred eating grain and corn and the availability of an open body of water. In addition, one group of geese remained in the city of Munich during the winter, benefiting from supplementary food from people. This information helps us to understand the different requirements of resident greylag geese. It can be used to inform management decisions aimed at preventing agricultural damage. Special goose areas can for example be designated for the different periods in a goose’s life cycle.

## Introduction

In several countries in Europe, including Ireland [1], Scotland ([2]; [3]), Germany ([4]; [5]), Britain as a whole ([6]; [7]; [8]), and the Netherlands ([9]; [10]; [11]), the greylag goose (*Anser anser*) was re-established during the 20^th^ century after native greylag geese populations had declined. These released greylag geese have formed new feral flocks. Over the last 30 years, the numbers of resident greylag geese have increased by up to 20% a year [11].

The increasing number of geese has led to a rise in geese-related problems requiring intervention. Countries with large resident geese populations like the Netherlands, Sweden, Norway, the USA, Great Britain and Germany have all reported problems such as foraging damage in agriculture ([12]; [13]; [10]), pollution in recreational centers ([14]; [15]), collisions with airplanes [16], the degradation of vegetation in agricultural and natural reserves ([14]; [17]; [18]), and the eutrophication of lakes and rivers ([14]; [19]).

Information on the habitat use and movements of resident greylag geese has been published in the Netherlands ([9]; [20]; [11]; [21]), Scotland [22] and Germany [23], among other countries, and studies of resident Canada geese (*Branta canadensis*) have been carried out in Nebraska [24]. Studies of neck-banded migratory greylag geese have been conducted in Sweden ([25]; [26]; [27]) and France [28].

In Germany as in other European countries, an increasing number of resident greylag geese have settled in new habitats over the last 30 years ([5]; [29]). This settlement has led to a rise in complaints and monetary losses affecting farmers, the tourist industry, airports and natural reserves. Governments must thus devise new management strategies to deal with these rising greylag goose populations. Most problems are encountered when the geese gather. This is especially the case during the breeding and rearing seasons and molting (April to July), and from November to January in the winter habitats. During the rest of the year the geese move more often and only rarely cause damage.

One such region subject to an increase in complaints and that has suffered monetary losses in agriculture amounting to approximately 50,000 Euro per year is the Altmuehlsee lake in Bavaria, an area of 12 km^2^ [30]. Since 1981, rising numbers of resident greylag geese have been observed in this region ([31]; [30]). The Altmuehlsee lake region is currently the largest breeding area in the south of Germany. Annual decreases in the numbers of resident greylag geese in summer after molting, and increases in February and March ([31]; [30]), are to be observed in several areas around the lake. This suggests that large numbers of the greylag goose population at the Altmuehlsee lake also leave their breeding and molting habitats after rearing their young and molting [31].

We fitted resident greylag geese families at the Altmuehlsee lake with satellite transmitters in order to investigate their movements and the habitats the birds use in summer and winter. The aim was to find out what it is in the breeding habitat or different structures in the winter habitat that causes most of the resident greylag geese to leave their breeding habitat after rearing and molting. Telemetric data can provide detailed information about the habitat used and movements of resident greylag geese, which can be used to help devise strategies to reduce monetary losses and the number of complaints. Additionally, the telemetric data gives a broader overview of the movements of the geese and thus also a deeper insight into their habitat needs. Data on the movements of the geese to foraging sites can help us to understand the structure of geese habitats. An awareness of the habitat needs of the geese and an understanding of the typical structure of geese habitats are basic requirements for the formation of management strategies. The objectives of our study were thus 1) to investigate the size of the home ranges and why the greylag geese leave the Altmuehlsee lake and 2) to determine which winter habitats the greylag geese use after leaving the lake in August.

## Materials and methods

### Study area

**Fig 1:**
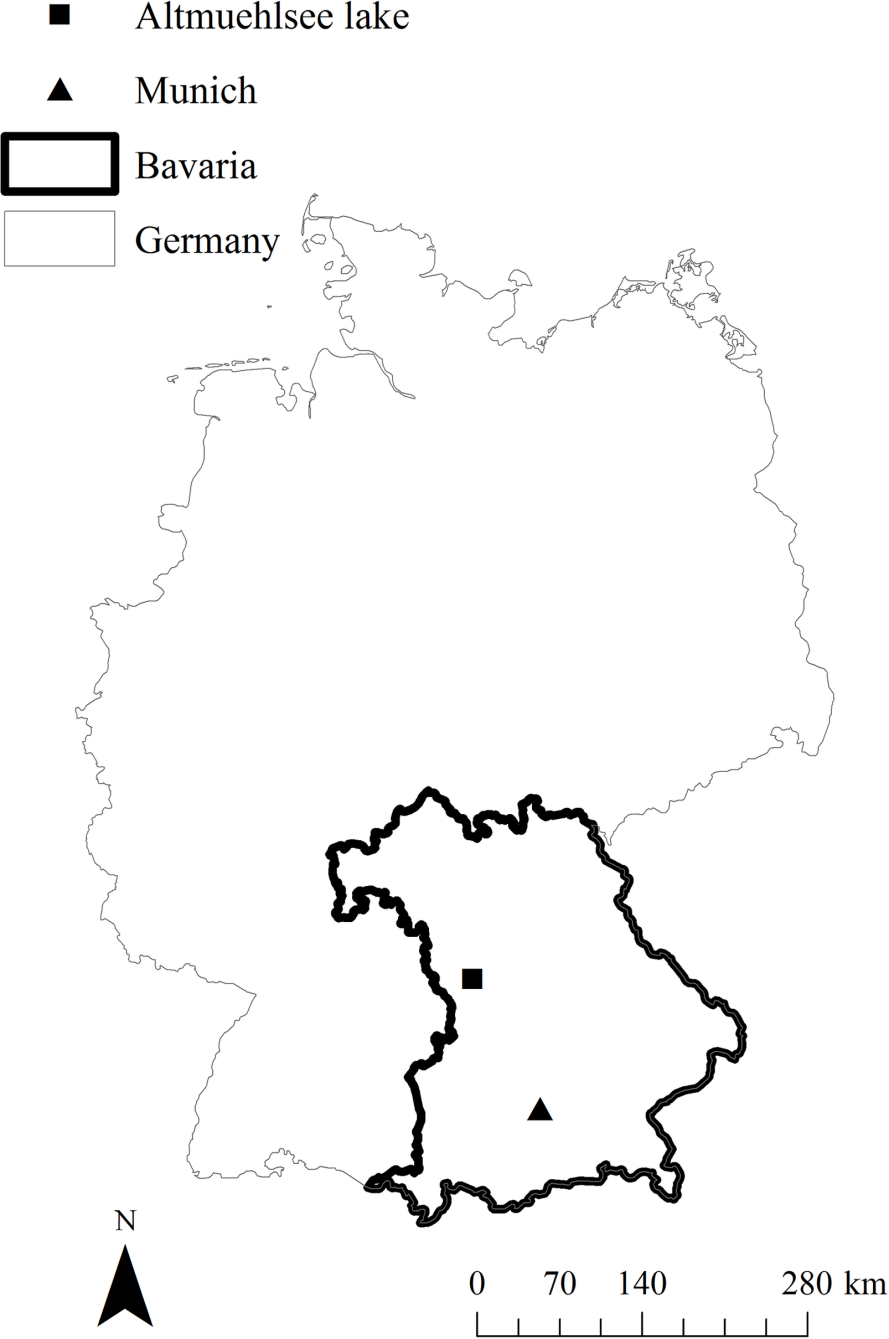
Map of Germany showing the position of the study area. Made with Natural Earth. Free vector and raster map data @ naturalearthdata.com.

In 2010 and 2011, we captured resident greylag geese at the Altmuehlsee lake (49° 7' 59" N, 10° 43' 29" O, 415 m above mean sea level) in Germany in their breeding and molting habitat. The Altmuehlsee lake is in Bavaria, southern Germany, 50 km south west of the city of Nürnberg (Fig 1). The artificially built lake was created in 1985 as a buffer between the arid north and the wet south of Bavaria. The Altmuehlsee lake is 4.5 km^2^ in area and is situated in an agricultural area, surrounded by 25% forest, 10% settlement and 65% agriculture (within a 5 km radius) (). Directly adjacent to the lake are meadows that are surrounded by hayfields and cropland, specifically corn, wheat, rapeseed and barley. To the west of the lake lie 1,100 ha of wetland habitat consisting of wet meadows. Twenty-eight percent of the lake consists of a 1.25 km^2^ protected area, made up of groups of islands and shallow water regions within the lake (88% water, 4% meadows, 1% sand, 1% reed and 6% bushes). Only 4% of the protected area may be entered by humans, and the rest remains completely untouched the whole year, creating an attractive habitat for waterfowl.

### Study design

During the flightless period of the goslings and the molt of their parents, which occur at the same time, we carefully drove resident greylag goose families feeding on meadows next to the lake into established fenced-in areas [32]. To ameliorate suffering, all fenced-in areas included shady parts and goslings as well as parents were released immediately after handling. In 2010 and 2011, we banded all caught greylag geese with the standard leg rings of the Radolfzell ornithological station and fitted 38 adult greylag geese with GPS (global positioning system) transmitters (Vectronic aerospace, Berlin). The transmitters were affixed as a backpack with a nylon harness. In addition to the transmitters, we banded these adults with a white leg ring with alphanumeric black numbers from the company Ecotone (Z50 to Z99). We used two types of battery-powered satellite transmitters: GSM (Global System for Mobile Communication) and VHF (very high frequency) models. GSM transmitters do not have external antenna but do have SMS (short message service) modules, and weigh 98 g. VHF transmitters have external antenna running parallel to the back of the goose. They do not have SMS modules, and they weigh 84 g. We used the heavier GSM transmitter for larger and heavier, adult greylag geese, and the lighter VHF transmitter for smaller greylag geese. We thus remained below the limit of 3% of body weight [33]. Both transmitters can take a maximum of 1,500 to 2,000 GPS points. We programmed both types of transmitters (GSM and VHF) to take positions every six hours (12:00, 18:00, 24:00, 6:00) from June to December to obtain information about wintering routes and habitats. Twice a week (Wednesday and Saturday) we took hourly positions for more detailed information about the behavior of the greylag geese.

Data from the VHF transmitters were downloaded via hand-held scanner and antennas twice a week between 12:00 and 14:00 (the period of time when VHF transmitters were online). After the geese had left the lake and settled in a new area, we visited the staging or wintering areas to check the fit of the transmitters and search for VHF modules every month.

We equipped 17 adult resident greylag geese in 2010 and 13 adults in 2011 with GPS (global positioning system) transmitters. Data on 19 of the 30 greylag geese with transmitters were included in our calculations (complete data for nine geese and partial data for 10 geese (Table 1)). Three of the 30 greylag geese were excluded from the analysis because they were one of a pair, and another eight birds were excluded because they lost their transmitters too early or were shot. Twelve resident greylag geese reached the winter habitat, five stayed in Munich, five were in the countryside area and two spent time in both habitats. All of the greylag geese bred and reared their goslings at the Altmuehlsee lake.

**Table 1:** Data for 30 greylag geese fitted with transmitters at the Altmuehlsee lake in Germany between 2010 and 2011.

ID	Period of tracking		Trans-mitter	Points Total	Distance [km]	Used for Analysis	End Cause
	Beginning	End					
**88490**	11 June 2010	06 July 2010	GSM	126	0	no	lost sender
**88530**	10 June 2010	29 July 2010	VHF	600	0	AS1	unknown
**88540**	10 June 2010	31 Aug 2010	VHF	1176	0	AS1, AS2	lost sender
**88550**	10 June 2010	07 Apr 2011	GSM	273	119	no	lost sender
**88560**	10 June 2010	22 July 2010	GSM	143	0	no	lost sender
**88580**	11 June 2010	17 Aug 2010	GSM	174	125	no	lost sender
**88610**	11 June 2010	27 June 2010	GSM	109	0	no	lost sender
**88620**	24 June 2010	03 Aug 2010	GSM	140	0	no	lost sender
**88630**	24 June 2010	29 Jan 2011	GSM	337	125	Munich	shot
**88500**	11 June 2010	30 Sep 2010	VHF	727	129	AS1	unknown
**88510**	11 June 2010	18 July 2010	VHF	812	0	AS1, AS2	unknown
**88520**	10 June 2010	16 Aug 2010	VHF	1300	17	AS1, AS2	unknown
**88640**	11 June 2010	08 July 2010	VHF	447	0	AS1	lost sender
**88650**	10 June 2010	18 Jun 2010	VHF	187	0	no	lost sender
**88660**	10 June 2010	18 July 2010	VHF	512	0	AS1	unknown
**88670**	11 June 2010	23 Jun 2010	VHF	288	0	no	unknown
**88680**	11 June 2010	04 Nov 2010	VHF	1132	129	AS1, AS2, Munich	unknown
**8849**	10 June 2011	10 Dez 2011	GSM	1233	138	AS1, AS2, Munich	sender crashed
**8854**	10 June 2011	11 Aug 2011	VHF	590	123	AS1, AS2, Munich	unknown
**8856**	09 June 2011	24 Apr 2013	GSM	1351	128	all	battery failed
**8858**	10 June 2011	08 Feb 2012	GSM	1068	38	AS1, AS2, countryside	died
**8861**	09 June 2011	22 Jun 2011	GSM	134	0	no	died
**8862**	10 June 2011	08 Nov 2011	GSM	1414	131	AS1, AS2, Munich	battery failed
**8858**	10 June 2011	08 Feb 2012	GSM	1068	38	AS1, AS2, countryside	died
**8861**	09 June 2011	22 Jun 2011	GSM	134	0	no	died
**8862**	10 June 2011	08 Nov 2011	GSM	1414	131	AS1, AS2, Munich	battery failed
**8863**	09 June 2011	07 Aug 2011	GSM	489	16	no	shot
**10090**	10 June 2011	19 Jun 2011	GSM	88	0	no	died
**10094**	10 June 2011	24 Sep 2012	GSM	1208	133	AS1, AS2, countryside	battery failed

(“used for analysis”: locations for which the geese delivered enough points to calculate statistics; distance: total distance from breeding site to wintering site)

### Data analysis

We excluded all GPS positions taken with fewer than four satellites (defined as “2D” by Software GPS-Plus (VECTRONIC Aerospace GmbH 2009), ensuring that we had an accuracy of < 4 m for each point. In total we deleted 224 GPS positions (from 21,019 positions, 1.07%). We used GPS-coordinates taken hourly for calculating distances geese moved to foraging sites. These distances were calculated as great-circle distances. The great-circle distances were used to test for differences between the distances the greylag geese flew at different times of day. The times of day were grouped as follows: morning (from 4:00 to 9:00), midday (10:00 to 14:00), afternoon (15:00 to 20:00) and night (21:00 to 3:00).

We assigned the data into four locations for all calculations: AS1 was the time geese spent at the Altmuehlsee lake during molting (breeding/molting area, June/July), and AS2 was the time geese were at the Altmuehlsee lake after molting (breeding/molting area, mid-July/August). The winter habitats were divided into “Munich”, which was the time the greylag geese remained in the city of Munich (winter habitat, September to December) and “countryside”, which was the time the greylag geese stayed in the “countryside” (area outside Munich, south of the Altmuehlsee lake) (winter habitat, September to December). Through daily observations we could identify the exact date of the end of the molt for each greylag goose, and grouped the data from that date on into AS2. Once the greylag geese left the Altmuehlsee lake without returning, we used the data following this date to identify winter habitats. We required a minimum of 140 GPS points for each place and goose for home range calculations; we thus did not consider staging areas where the greylag geese stayed for less than 4 weeks.

We used Program R (V. 3.0.2) [34] for statistical analysis. Home range calculations included time to statistical independence (TTSI) [35], site fidelity, Minimum Convex Polygon (MCP, 95%), fixed Kernel Density Estimation (KDE, 95%) to identify special habitats [36] with Least Square Cross Validation ([37]; [38]) and calculations of an asymptote for each animal and region [39]. Home range data were considered only when the home range approached an asymptote and when TTSI and site fidelity were reached [38]. KDE 95% was chosen to compare the results with literature, as it shows only the places that the resident greylag geese truly use. MCP 95% was chosen to get a basis for calculating habitat structures to explain differences in the distances the greylag geese flew to foraging sites.

We identified the types of habitat situated in the home ranges by importing the calculated MCPs into ArcMap [40]. We computed the percentage of area used (agriculture, water, island, forest, anthropogenic area) for each resident greylag goose using land use-data. For further calculations, we grouped the data into “areas used” (islands, water and agriculture) and “areas unused” (anthropogenic area, forest and groves). All types of habitats lie within the home range. However, “areas unused” were areas for which no GPS points were found, suggesting that geese do not use them.

The data are not considered statistically independent, since we took several observations and locations for each goose. We therefore had to use a mixed effects model for comparison of distances to foraging sites and a linear effects model for the home range sizes (KDE 95%). To test the need for random effects for both cases, we used two methods, a comparison via ANOVA of a null model (linear model) without the random effect (individual) and a full model (linear mixed effects model) with random effect (individual) and additionally a likelihood-ratio test.

Using the R package “nlme” to “fit and compare Gaussian linear and non-linear mixed-effects models” [41], we calculated the relationship between home range size and location with the fixed effects "location" and "areas unused". According to our tests, the random effect “individual” should not be considered. (form of the model: h*ome range size* = *β*_0_(*location*) + *β*_1_(*area unused*) +*ε*).

The relationship between distances to foraging sites (log transformed) to time period and time of day was examined by applying a mixed effects model. We used location and time of day as fixed effects and the individual (ID) as the random effect to account for repeated measurements of the individual marked goose (random effect should be considered according to ANOVA and likelihood-test) (form of the model: log(*distance*) = *β*_0_(*location*) + *β*_1_(*time of day*) + *ID* + *ε*). For a paired comparison of all independent variables within the model, we used the R package “multcomp” calculating “simultaneous tests and confidence intervals” [42] and the Tukey post-hoc test. Collinearity, influential data points, lack of normality and homoscedasticity could be precluded by visual inspection of residual plots for each model. To evaluate the relationship between the size of the home range and the distance geese flew to foraging sites, we used Pearson's product-moment correlation.

We calculated the distances from each GPS point on the foraging site to the nearest water. Additionally, we analyzed the habitat the geese fed on, determining whether it was a field, a meadow or grass next to the lake, and calculated the proportion for each region.

#### Ethic statement and permissions

All geese were collected according to German Regulations. The permission to catch, ring and fit geese with GPS transmitters were given by the government of Middle Frankonia (Mr. Nagel, 55.1–8642 G 022/09, Date 7.4.2009). The government of Middle Franconia together with the District office of Gunzenhausen approved and supervised animal welfare.

## Results

### Home range and habitat

Mean home range size (Fixed Kernel Density Estimation 95% (95KDE)) at the Altmuehlsee lake during the flightless period of the greylag geese was 2.9 ± 3.1 km^2^ (±: SD), increasing to 9.4 ± 6.5 km^2^ for 95KDE when the greylag geese could fly again. The above difference was however not statistically significant (*F*_*4*,*39*_ = 27.5, *n* = 31, *P* = 0.092). In this calculation the larger home range found after molting (AS2) contained more area that the greylag geese did not use (“area unused”) but flew over. However, home range calculations using KDE (95%) include the 95% closest points and unite all space around the points. That also includes habitats like forest or anthropogenic areas and shows that the greylag geese used places lying next to such habitats. We also found that the habitats the greylag geese used within the home ranges differed between the two time periods at the Altmuehlsee lake. When the greylag geese molted at the Altmuehlsee lake (AS1), they did so on water (43% of the time) and on islands, agricultural land, forest land and in anthropogenic areas for 8%, 40%, 3% and 6% of the time respectively. After the molt at the Altmuehlsee lake (AS2), less water habitat (26%) and more agricultural (54%) and anthropogenic (11%) habitats were found in the home range.

**Fig 2:**
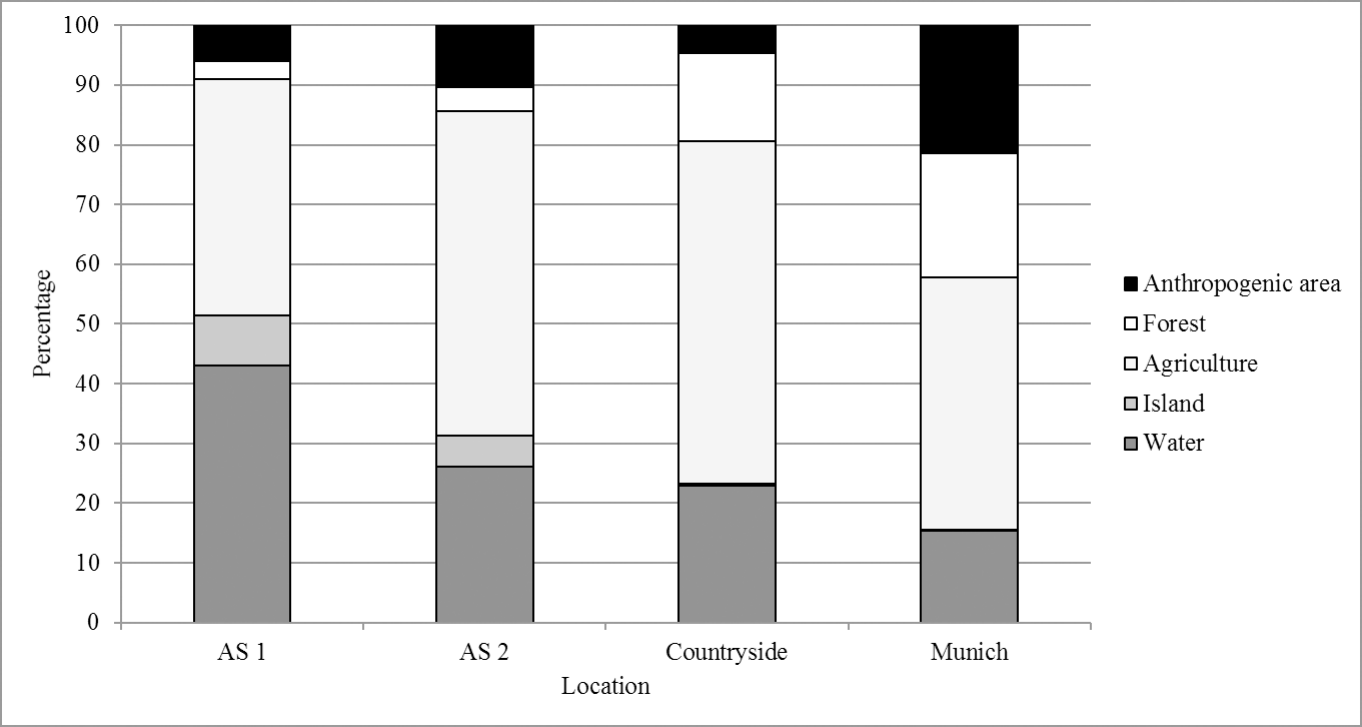
Average percentages of habitats presented in the home ranges at the Altmuehlsee lake and in the wintering areas. (AS1: during molt, AS2: after molt), in the countryside and in the city of Munich.

In the winter habitats, the greylag geese used smaller home ranges (4.0 ± 2.3 km^2^ for 95KDE) (*F*_*4*,*39*_ = 27.5, *n* = 19, *P* < 0.001) in the countryside and in the city of Munich (3.3 ± 1.6 km^2^ for 95KDE) (*F*_*4*,*39*_ = 27.5, *n* = 19, *P* < 0.001) compared to the time after molting at the Altmuehlsee lake (AS2). In the countryside, the habitats within the home range showed fewer anthropogenic areas (5%) but more forest area (15%) than at the Altmuehlsee lake (AS2) (Fig 2). In the city of Munich, there was less water (15%) and agricultural land (42%) but more forest (21%) and anthropogenic areas (22%) than in the countryside (Fig 2) (*χ*^2^ = 4, *df* = 4, *P* = 0.406). There were no or few small islands in the wintering areas compared to the breeding areas.

### Movements of the greylag geese

In each of the locations, the resident greylag geese used one resting site and one to ten foraging sites. The greylag geese covered a mean distance from their resting site to foraging sites during molting at the Altmuehlsee lake of 292.9 m (max 1,900 m, min 50 m) (AS1). They fed only on grass next to the water (99.4%), with a mean distance of 17.7 m from the water (max 165.1 m, *n* = 2177). To reach the feeding sites the greylag geese had to cross the water between the resting site (island) and the feeding site. After molting, the greylag geese increased the distance travelled to foraging sites to 782.0 m ± 313,4 m (*n* = 2004, *P* < 0.001) and used more fields (38.2%) and meadows (21.8%) for feeding (AS2). On average, the resident greylag geese tried to remain within 121.7 m of the water (mean distance 121.7 m, max. 870 m). In the winter habitats in the countryside, the greylag geese flew a similar distance to foraging sites (mean distance 1,582.6 m, *n* = 778, *P* = 0.985) compared to the Altmuehlsee lake (AS2). However, they fed on more fields (58.4%) and also on grass next to the water (32%). These foraging sites were situated within 186.1 m of water. In the city of Munich, the greylag geese flew shorter distances to foraging sites compared to the Altmuehlsee lake (mean distance 219.7 m ± 1150.6) (*n* = 883, *P <* 0.001). They fed more on grass right next to the water (65.9%) and on meadows not directly at the water (25.8%), but stayed a maximum of 76.2 m away from water during feeding (Table 2).

**Table 2:** Percentage of usage of the different feeding habitats used by greylag geese in the four locations, as well as the average and maximum distances geese fed from the water.

Location	Type of habitat	Percentage of usage	Average distance to water [m]	Max. distance to water [m]
**AS1**	grass next to water	99.4	17.7	165.1
	field	0.1		
	meadow	0.5		
**AS2**	grass next to water	40	121.7	870.7
	field	38.2		
	meadow	21.8		
**countryside**	grass next to water	32	186.1	1,540.2
	field	58.4		
	meadow	9.6		
**Munich**	grass next to water	65.9	76.2	852.8
	field	8.3		
	meadow	25.8		

Additionally, in comparing the size of the home range and the distance the resident greylag geese flew to foraging sites, we found increased distances travelled to foraging sites with an increase in size of home range. The increased size of the home range was due to an increase in the unused area in the home range (Pearson's product-moment correlation: t = 4.7, df = 57, *P* < 0.001, r = 0.529).

**Fig 3:**
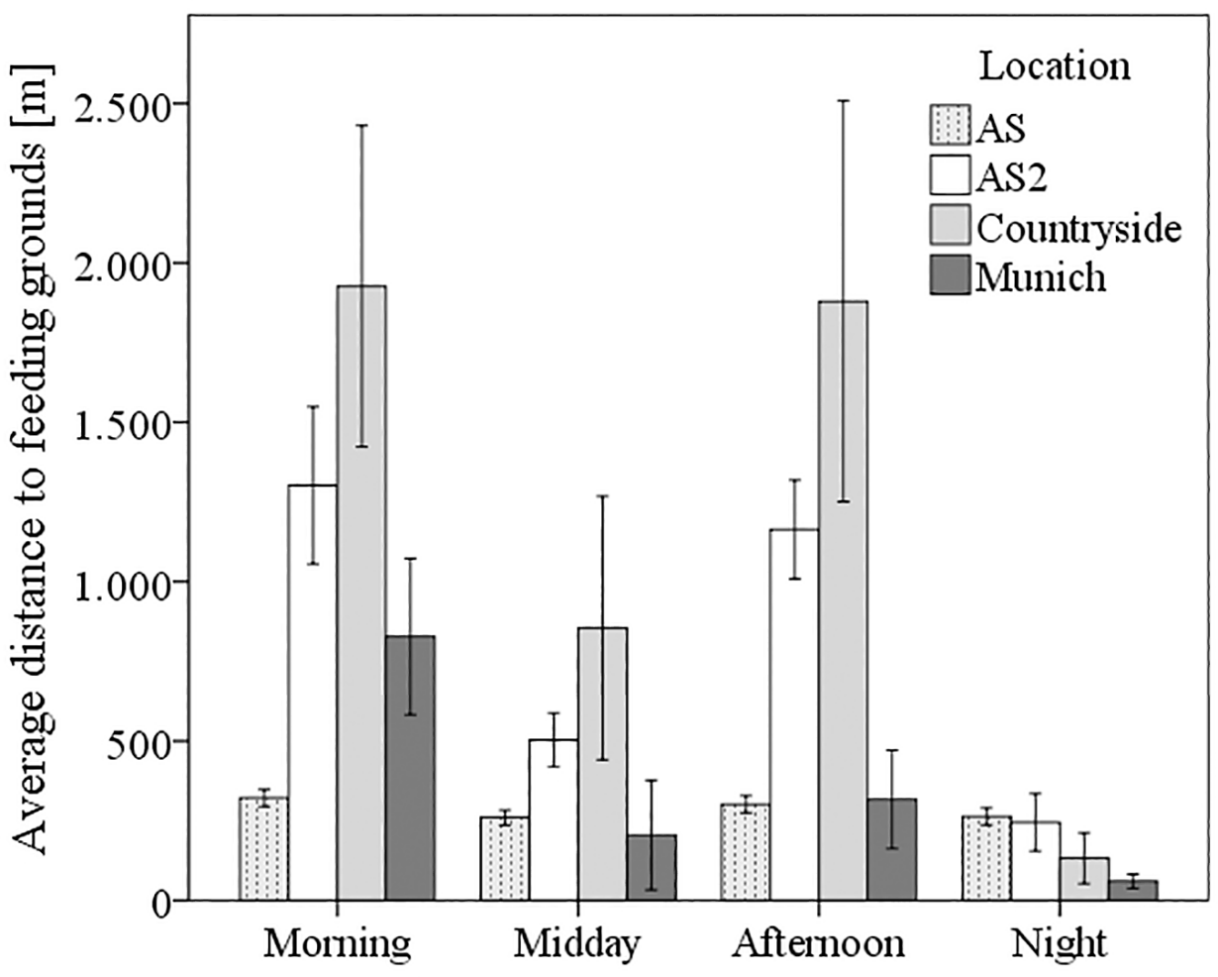
Average distances in meters that greylag geese flew to their foraging sites in the four habitats. (AS1: the Altmuehlsee lake during molt, AS2: the Altmuehlsee lake after molt, Countryside, Munich) during four different periods of the day. (Error bar: 95% CI).

Generally, the resident greylag geese flew longer distances to the feeding grounds in the morning and evening than around midday and during the night. Typically, the greylag geese left the resting site to fly to one of their foraging sites once in the morning and once in the evening. If they flew out to feed at midday or at night, they used nearer feeding grounds than they tended to during the morning and evening. Statistically differences were recognized in the winter habitats in that the distances the greylag geese moved in the morning (between 828 m (Munich) and 1,926 m (countryside)) and afternoon (between 317 m (Munich) and 1,879 m (countryside)) were longer than at midday (between 205 m (Munich) and 854 m (countryside)) and at night (between 60 m (Munich) and 132 m (countryside) (*P* < 0.002) (Fig 3). The distances travelled during the night did not exceed a maximum of 245 m (at the Altmuehlsee lake) (*P* = 0.138, *n* = 2641).

### Seasonal movements

**Fig 4:**
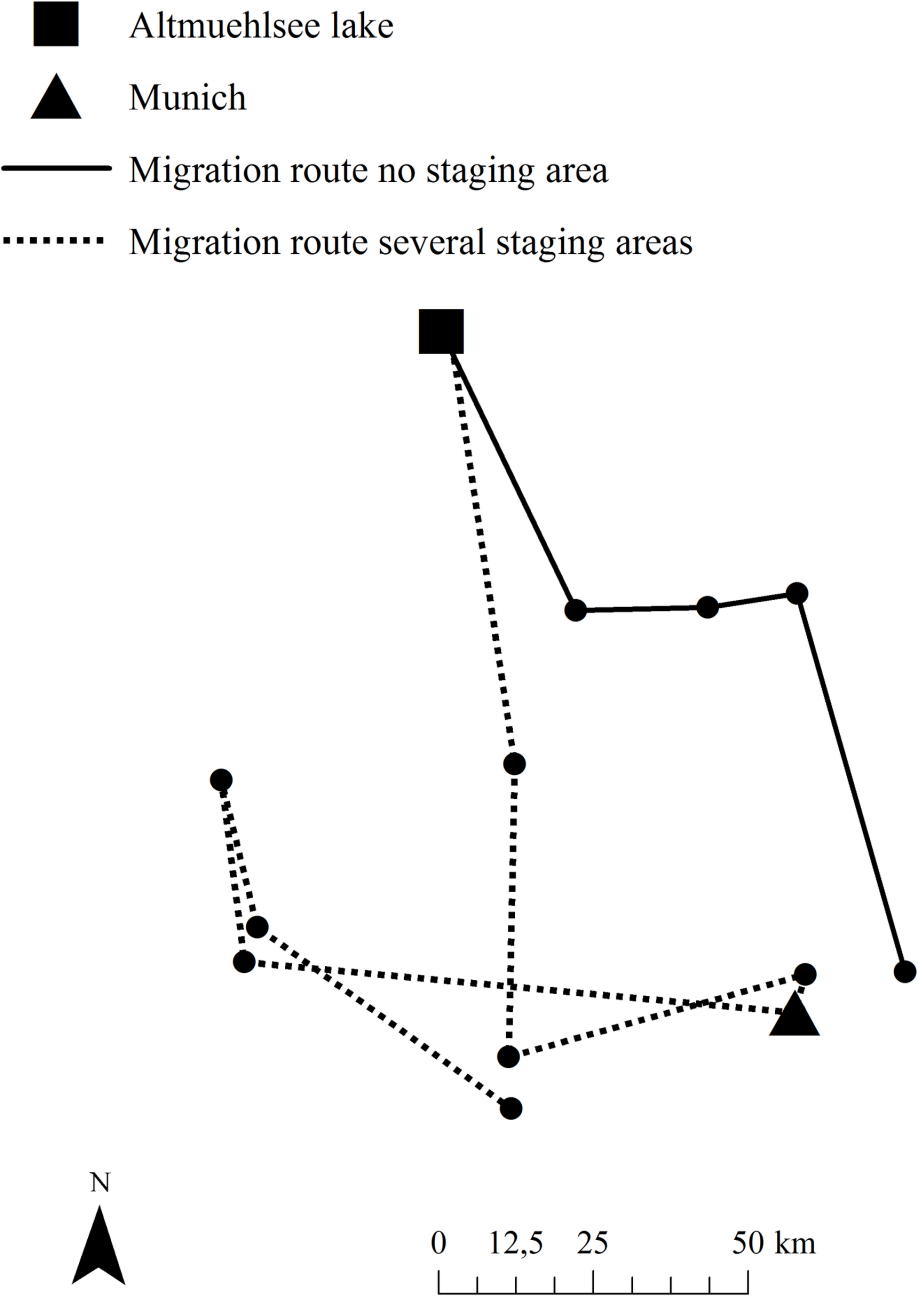
Map showing the study site and two routes of marked geese to wintering sites.

All resident greylag geese with transmitters left the Altmuehlsee lake shortly before or after the start of the hunting season (1st of August) and followed the River Altmuehl to the south. The greylag geese (*n* = 12) stopped for an average of 9.4 days (min = 7 hr, max = 25 days) at the River Danube. The greylag geese then moved between 13 and 73 km (the total distances from the breeding habitat to the winter habitat ranged from 73 to 133 km) to their winter habitats (Fig 4). Seven of the 12 greylag geese used the River Danube as their only staging site, two greylag geese used one additional stop, and three greylag geese used seven or eight staging areas up to the end of November. The greylag geese with several staging sites remained a mean time of 18.7 days at each site. Starting at the end of November or the beginning of December, all greylag geese remained in one wintering location until they left the winter habitats to return to their breeding habitats in February.

For wintering sites, the greylag geese used the city of Munich as well as several areas south of the Altmuehlsee lake (countryside). The countryside area stretches from 48° 45' 42" N, 11° 47' 45" E to 47° 59' 23" N, 10° 15' 00" E (Fig 4). All of these locations consist of a lake or a river with islands or shallow water surrounded by agricultural land, forests and, depending on the region, towns. The city of Munich (48° 8' 14" N, 11° 34' 31" E, 515 m, area: 310.71 km^2^) has several parks with lakes surrounded by meadows and small forests. There is very little agriculture in the inner city, but there are agricultural areas on the outskirts.

## Discussion

During molting and the rearing of their young, geese prefer to stay close to the water, which serves as a refuge in case of danger. Feeding grounds close to the lake consist mainly of meadows. Consequently, home ranges are rather small and contain predominantly water, islands and to some extent agricultural areas. Forest or anthropogenic areas are avoided during this time. A similar habitat structure for greylag geese was also described by Klok *et al*. [10] in the Netherlands. The home range size of 2.9 ± 3.1 km^2^ during molting at the Altmuehlsee lake is comparable with that reported in other studies. Dunton and Combs [43] found home ranges up to 1.1 km^2^ for giant Canada geese (*Branta canadensis maxima*), Hughes *et al*. [44] found 1.8 km^2^ home ranges for greater snow geese (*Chen caerulescens atlantica*) and Lewis *et al*. [45] found 4.3 km^2^ home ranges for black brant (*Branta bernicla nigricans*). Home range sizes for migratory geese during the winter ranged from 4.3 km^2^ for barnacle geese (*Branta leucopsis*) [46] to 6.1 km^2^ for brant geese (*Branta bernicla*) [47], which is in line with our results (4.0 km^2^ in the countryside and 3.3 km^2^ in the city of Munich).

When our greylag geese were able to fly again after molting they used different habitats at the Altmuehlsee lake. They used more harvested grain and corn fields and more meadows around the lake. Because the greylag geese can fly again after the molt, they can reach fields that are away from the water. While home range size increases, the size of the area used by the greylag geese remains almost the same, because the greylag geese use the island in the middle of the lake as a resting site from which to fly in all directions to foraging sites. On their way to the foraging sites, or next to their foraging sites, they cross more forests or anthropogenic areas, such as towns, farms and forests. As the new foraging sites are far from the resting site, the distance the greylag geese fly to these places increases. From June to August, approximately 1,500 geese stay at the lake [30] for molting and breeding. The proportion of grain and corn fields is quite low in this region, as large parts of the region are covered with meadows. The greylag geese therefore have to fly further distances to foraging sites, as they prefer grain and corn fields. Amat [48], Patterson *et al*. [49] and Giroux and Patterson [50] found that greylag geese and pink-footed geese (*Anser brachyrhynchus*) fly similar distances to foraging sites, flying between 100 m and 5 km respectively. When they wanted to reach specific fields, they increased their distances up to 5 km ([48]; [50]). Bell [51] found that geese fly increasing distances to foraging sites when larger groups are observed. We observed the same result in our study, especially at the Altmuehlsee lake.

From the end of July to the beginning of August, the observed greylag geese leave the Altmuehlsee lake for locations where they stay throughout the winter. In contrast to our observations, van Turnhout *et al*. [20], Voslamber [11], Voslamber *et al*. [21] and Maarten *et al*. [9] reported that resident greylag geese in the Netherlands remained sedentary at one location throughout the entire year. However, they also found resident greylag geese that moved up to 200 km, especially during fall and winter.

As most of the resident greylag geese leave the Altmuehlsee lake in August and return in February and March ([31]; [30]) it is possible that the site lacks something that they need in winter, so that it is not suitable for the greylag geese to remain there. To obtain the same amount of energy from grass as from grain, geese have to take in 10 times more grass [26]. This fact and the low amount of grain and corn available at the Altmuehlsee lake might explain why the tested greylag geese leave the lake in August. By August the greylag geese have consumed large parts of the fields, and the only remaining food available is grass. Another factor explaining why the geese leave the lake could be the start of the hunting season on the first of August. The determining factor cannot be proved with the data from our study. However, it is also possible that something is lacking in the winter habitats that the greylag geese need for breeding. In the city of Munich, the goose population density is much higher than at the Altmuehlsee lake (own observations). Clutch mapping programs have established that there are also fewer places for breeding available in the city area. It has been observed that nests are used several times at once and the distances between the nests are very short [29]. This indicates increased stress for the geese in Munich, so that only stress-resistant animals start breeding there. The other geese look for places to breed where they are subjected to less stress, such as the Altmuehlsee lake. (Confirmed by sightings of greylag geese at the Altmuehlsee lake that had been ringed in Munich.)

The start of the hunting season on the first of August might also be a reason for the greylag geese to leave the lake. It has been shown that geese change their foraging sites and behavior as well as their movements when hunting occurs [29]. However, the hunting season for greylag geese starts on the first of August throughout southern Germany, with a similar intensity in all regions where greylag geese live except for the city of Munich. Some of the graylag geese will be attracted to the city of Munich area by the availability of supplementary food [29] and lack of a hunting season. In the countryside area hunting does occur. The more abundant supply of corn and grain on agricultural land might compensate for the disturbances caused by hunting.

The structures of the winter habitats are similar. They also include a river or a lake with fields around it. Though the resident greylag geese use smaller home ranges in their winter habitats, the distances to the feeding grounds are not shorter. As in the research by Bell [51], who found that greylag geese in Scotland stayed at some fields throughout the winter, our greylag geese fed at one to four foraging sites, between which they switched regularly. These sites are not far from each other and all are in the same direction from the resting site. As geese prefer grain and corn, and large fields with these foods are available in these sites, the greylag geese benefit by not having to spend much time searching for other food. Additionally, the number of geese in the winter habitats does not exceed 60 to 100 geese. Competition for food is thus much lower and the amount of food lasts longer. This makes it possible for the greylag geese to use only four fields and one resting site during the whole winter. At the Altmuehlsee lake, the greylag geese use up to 10 foraging sites that lie in all directions from the resting site. The greylags had to switch foraging sites frequently because the fields were quickly exhausted. The greylags’ home range is thus extended to include up to 10 foraging sites all around the lake. The home ranges in the winter habitats stretch from the resting site to only four foraging sites lying in one direction, which results in a smaller home range. In total, having more foraging sites in all directions results in the geese having a larger home range, although the distances they fly to single foraging sites is similar.

Gauthier *et al*. [52] and Alisauskas and Ankney [53] found for greater snow geese (*Chen caerulescens atlantica*) and lesser snow geese (*Chen caerulescens caerulescens*) respectively that the geese exhibited higher feeding activities in the mornings and evenings, and more resting on water bodies during daytime and at night. Amat [48] and Nilsson [26] found the same for greylag geese, and the same behavior was also observed in our study group.

The greylag geese that stayed in Munich during the winter used a similar home range to the greylag geese in the countryside. The home range size in Munich is comparable with that of resident Canada geese (*Branta canadensis*) in a city in the USA [24]. As there are more houses and streets in the city of Munich, the home range contains larger areas of this type of habitat. The greylag geese used a park and the lake as resting sites and fed on the lawns around the park. They seldom left the park to go to other meadows or fields. All geese get much of their food, such as bread and oats, from tourists and residents. Sears [54], Ryley and Bowler [55] and Kässmann and Woog [56] showed that waterbirds get a large amount of their food in cities from humans in a shorter time than they would in a more natural setting. By obtaining food from humans, the birds save energy because they do not have to search for food. For this reason, the greylag geese do not have to fly as long distances to foraging sites as they do in the countryside. Furthermore, supplementary food helps the geese to survive in a location where natural energetic foods, such as grains and corn, are very rare. Supplementary food from humans can cause geese to change their behavior in the winter so that they remain in cities near people. Kässmann and Woog [56] found that a group of resident greylag geese in Stuttgart, Germany, adapted their behavior to food provided by humans. Additionally, the geese in Munich benefit from tourists and residents because they keep the lakes open in the winter and remove the ice, allowing the geese to remain there during the whole winter. Although the number of geese in some parks in Munich is higher, the supplementary food and higher temperatures in cities makes it easier for the geese to survive the winter.

We found that resident greylag geese in southern Germany use different types of habitats during the year and make seasonal movements. During the winter, they need high-energy food, such as corn and grain, or supplementary food, as well as an open body of water. At the Altmuehlsee lake, these resources are not available: the portion of corn and grain is restricted and the lake freezes up completely in the winter. Overall, the greylag geese used up to four foraging sites and one resting site. Additionally, having only a small number of geese in the winter habitat was beneficial. Hunting or human disturbances are accepted when the other factors are considered.

Our results show that resident greylag geese can adapt to different habitat types and make seasonal movements. In winter habitats, it could be helpful not to plough grain and corn fields after harvest in order to leave some food for the geese and thus prevent damage to newly sown fields in locations next to open bodies of water. For breeding and rearing habitats, islands, lots of water, and grassland next to the water are essential.

This information can be used to structure geese habitats and non-geese habitats, thus helping to direct the geese to certain areas, to reduce damage and to make it possible to manage increasing numbers of geese [30].

## Supporting information

S1 File Complete dataset used for analysis. (XLS)
